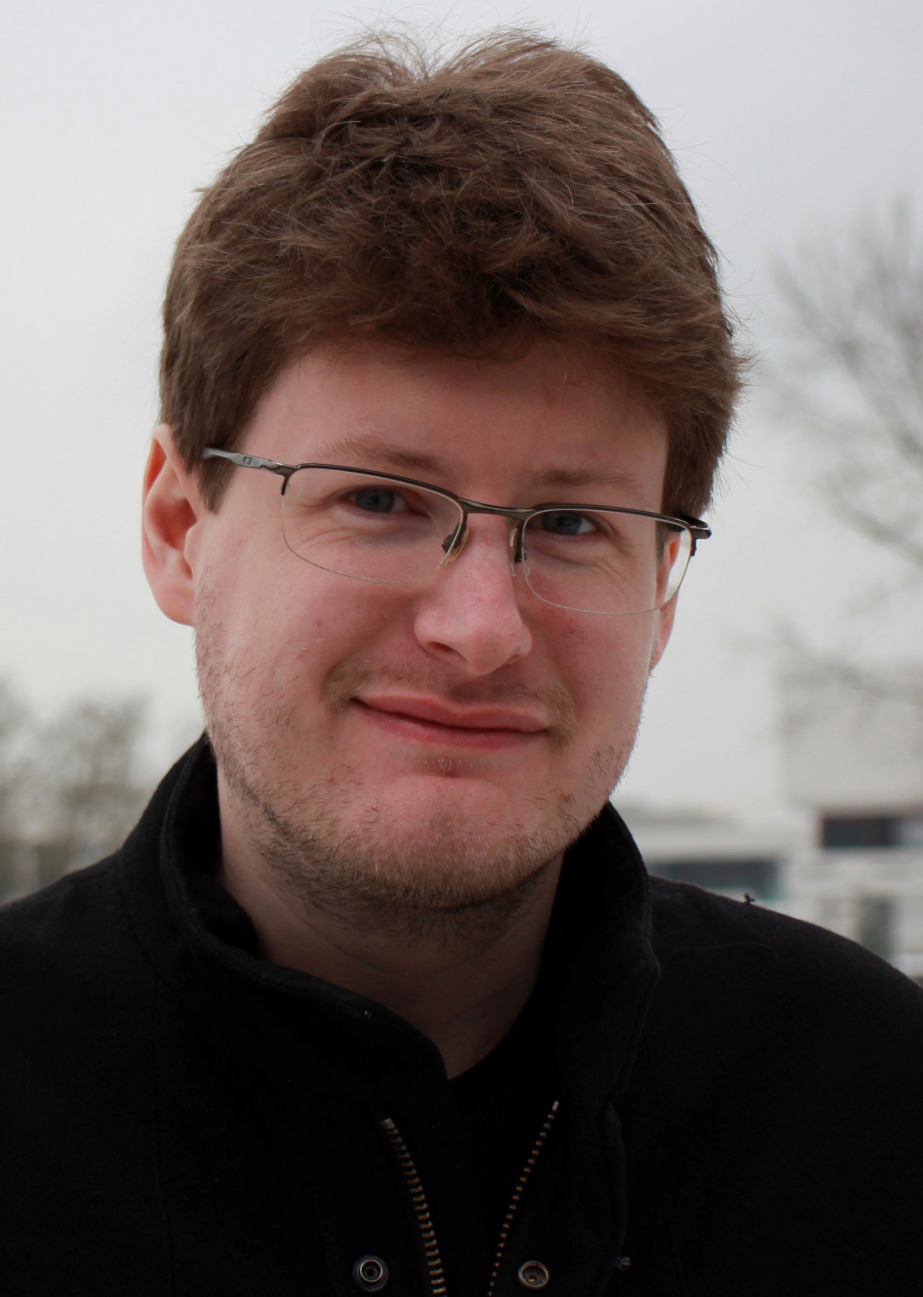# Recent Breakthroughs in P_4_ Chemistry: Towards Practical, Direct Transformations into P_1_ Compounds

**DOI:** 10.1002/anie.202205019

**Published:** 2022-05-25

**Authors:** Daniel J. Scott

**Affiliations:** ^1^ Department of Chemistry Chemistry Research Laboratory University of Oxford 12 Mansfield Road Oxford OX1 3TA UK

**Keywords:** Catalysis, Direct Transformations, Monophosphorus Compounds, Organophosphorus, White Phosphorus

## Abstract

For several decades, academic researchers have been intensively studying the chemistry of white phosphorus (P_4_) in the hope of developing direct methods for its transformation into useful P‐containing products. This would bypass the hazardous, multistep procedures currently relied on by industry. However, while academically interesting P_4_ activation reactions have become well established, their elaboration into useful, general synthetic procedures has remained out of reach. Very recently, however, a series of independent reports has begun to change this state of affairs. Each shows how relatively simple and practical synthetic methods can be used to access academically or industrially relevant P_1_ compounds from P_4_ directly, in “one pot” or even in a catalytic fashion. These reports mark a step change in the field of P_4_ chemistry, and suggest its possible transition from an area of largely academic interest to one with the promise of true synthetic relevance.

## Graphical abstract

The transformation of white phosphorus directly into useful P_1_ products has long been an extremely challenging synthetic goal. However, in the past few years, several independent breakthroughs have shown that such reactions are achievable using a variety of different mechanistic strategies. This Minireview highlights these recent innovations, discusses their key features, and analyses their implications for the future development of this field.

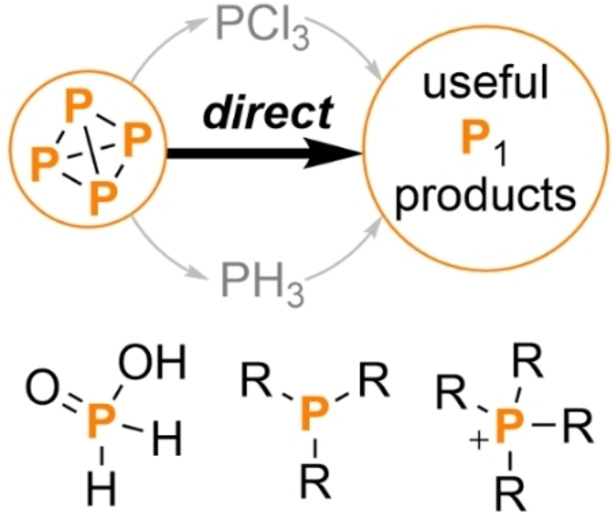

## 1 Background and Introduction

**Scheme 1:**
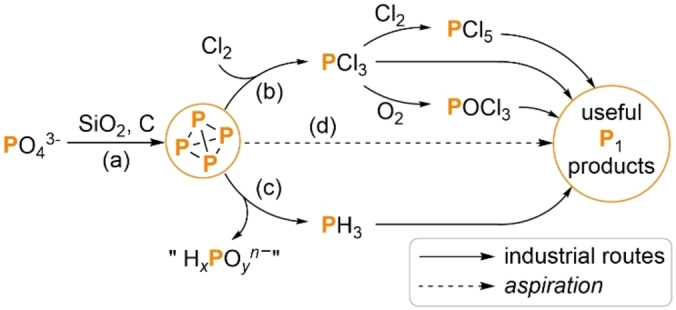
Current industrial synthesis of P_1_ compounds via reduction of phosphate ores to P_4_ (a) and subsequent transformation into PCl_3_ (b) or PH_3_ (c). Direct transformation of P_4_ into P_1_ compounds (d) remains highly challenging. H_*x*_PO_*y*_^*n*−^=H_3_PO_4_, H_2_PO_2_^−^ and/or HPO_3_^2−^.

White phosphorus (P_4_) is one of the single most important feedstock materials for the modern phosphorus chemical industry, being prepared on megaton scale each year via thermal coke reduction of phosphate (found in apatite ores) in electric arc furnaces (Scheme 1a).^[1]^ The majority of this P_4_ is re‐oxidized to generate high‐purity phosphoric acid and other phosphate materials, while the remainder is transformed into the enormous variety of other phosphorus‐containing products that are found throughout industry and academia.^[2]^ These include the broad family of organophosphorus compounds, which find uses in areas as diverse as photoinitiators, pharmaceuticals and flame retardants (among many, many others), and in all cases are prepared using P_4_ as their common P atom source.^[3]^

As a consequence, the methods that are used to transform P_4_ are extremely important. Unfortunately, the current industrial state‐of‐the‐art suffers from a number of significant and long‐recognised drawbacks. Probably the best‐known route is the direct reaction of P_4_ with toxic and oxidizing Cl_2_ gas to generate PCl_3_, a highly corrosive liquid that must be stored and transported under inert atmosphere in corrosion‐resistant containers (Scheme 1b).^[2c]^ PCl_3_ can be further oxidized to PCl_5_ or POCl_3_ using Cl_2_ or O_2_, respectively, and reaction of these electrophiles with suitable nucleophiles then furnishes the desired P_1_ products, alongside chloride‐containing waste byproducts.^[4]^ This route is often exemplified by the industrial preparation of Ph_3_P, prepared by reaction of PCl_3_ with PhCl and sodium metal.^[2d]^ Alternatively, P_4_ can be transformed through disproportionation under acidic or basic conditions to generate toxic and flammable PH_3_ gas, alongside oxidized P_1_ byproducts (Scheme 1c).^[5]^ This PH_3_ can then be used in the formal hydrophosphination of unsaturated substrates, via either radical or ionic mechanisms.[^2c^, ^2d^]

Clearly, both routes suffer from a reliance on highly hazardous reagents and intermediates, and other criticisms have included undesirable levels of waste formation and the multistep nature of these transformations. Recognition of these limitations has prompted intense interest in the development of alternative synthetic strategies, with a particular emphasis on the goal of achieving *direct*—and, if possible, *catalytic*—transformation of P_4_ into useful P‐containing products,^[6]^ thus bypassing the hazards, inefficiencies and increased practical complexity associated with the current, indirect methods.

As a result, the past several decades have seen extensive investigations by numerous research groups into the elementary reactivity of the tetrahedral P_4_ molecule, with a particular emphasis on its activation using (typically electron‐rich) *d*‐block metal complexes (and, to a lesser extent, *p*‐ and *f*‐block).^[7]^ Unlike many other industrially relevant inorganic small molecules (e.g. H_2_, N_2_), the challenge in P_4_ functionalization typically arises not because the molecule lacks kinetic reactivity,^[8]^ but rather from the difficulty in channeling its reactivity through a specific mechanistic pathway to cleanly provide a single product of interest. This reflects the fact that any transformation of the P_4_ tetrahedron into P_1_ products must necessarily achieve six separate P−P bond‐cleavage steps alongside many distinct P−E bond‐formation steps, all in a controlled manner. Phosphorus has also been referred to as “the carbon copy” due to its diagonal relationship and correspondingly similar chemistry, which allows for the generation of an extremely wide variety of stable P_*n*_ structural motifs.^[9]^ These include both saturated and unsaturated chains, rings and cages, with *n* less than, equal to, or greater than 4.^[10]^

As a result, it is perhaps unsurprising that many reactions of P_4_ can result in unpredictable and unselective outcomes. Nevertheless, the reaction of P_4_ with metal complexes has by now become well established and has proven a boon for the field of polyphosphorus coordination chemistry, providing access to a wide variety of fascinating and previously unprecedented structures.^[11]^ Unfortunately, despite extensive progress in such “activation” of P_4_, selective subsequent functionalization of the resulting P_*n*_ fragments has been far less explored, and remains extremely challenging.^[12]^ In particular, *release* of functionalized P_*n*_ fragments—and industrially relevant P_1_ fragments especially—has been reported only rarely.^[13]^ Alongside metal‐mediated processes, the reactivity of P_4_ towards other types of reagents such as simple nucleophiles and electrophiles has also been investigated, but again this has led to only limited successes. For example, investigations starting as early as the 1960s showed that organophosphorus derivatives could be prepared via reaction of P_4_ with organolithium reagents RLi, but with generally poor yields and selectivity.^[14]^

Until recently, the conspicuous lack of practical new synthetic methods for P_4_ transformation—in contrast to the wealth of new insights regarding the more fundamental reactivity of P_4_—might reasonably have prompted the pessimistic question of whether general, direct generation of commercially relevant P_1_ products from P_4_ is really an achievable objective. Fortunately, the past two and a half years have seen a sudden surge of progress towards this goal, with the appearance of a series of publications each describing the successful direct conversion of P_4_ into one or more P_1_ products. Notably, these reports have arrived independently, from separate research groups, and through pursuit of distinct mechanistic strategies. Collectively, these breakthroughs suggest that the goal of general, practical and direct P_4_ functionalization may finally be coming within reach.

In the following sections the key aspects of these recent innovations will be described and analyzed, and directions for the future development of this research field will be suggested. For the sake of focus, this Minireview will only describe examples in which P_1_ products can be obtained *directly* from P_4_: that is, in a single reaction without isolation or purification of any intermediate products (although the reaction itself may consist of several experimental steps). Similarly, given the specific importance and challenge of generating *organo*phosphorus compounds, discussion will be limited to systems that can be used to construct P−C bonds (although examples of other P−E bond formation using these same systems will also be described).[^15^, ^16^] Fortunately, several excellent, comprehensive reviews are available for readers who are interested in supplementing this Minireview with a broader overview of recent (and not so recent) P_4_ chemistry.[^7^, ^11^, ^12^]

## 2 Recent Breakthroughs

### 2.1 Photocatalytic Arylation of P_4_

**Scheme 2:**
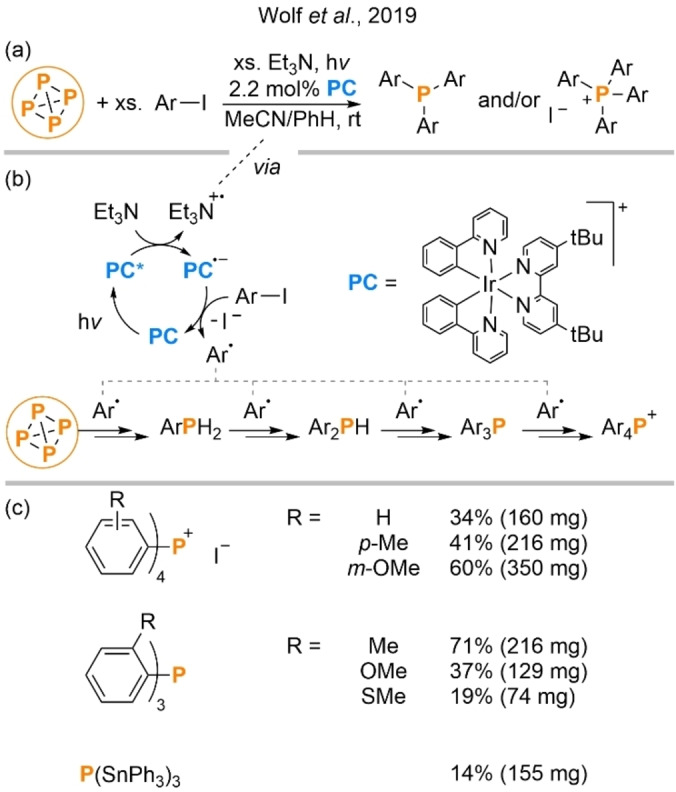
Direct photocatalytic arylation of P_4_ using aryl iodides (a), proposed mechanism (b) and isolated products (c). Products observed but not isolated are not shown. For P(SnPh_3_)_3_, Ph_3_SnCl was used instead of ArI. Catalyst loading in mol % is defined per P atom.

Arylated P_1_ derivatives are among the most industrially and academically important families of phosphorus‐containing compounds. Indeed, the simplest such derivative, Ph_3_P, is often invoked as a “benchmark” example of an important P_1_ product whose synthesis directly from P_4_ would be highly desirable. In late 2019 Wolf et al. reported that P_4_ can be directly transformed into triarylphosphines (Ar_3_P) and tetraarylphosphonium salts (Ar_4_P^+^) by treatment with aryl iodides (ArI) and Et_3_N in the presence of an Ir‐based photocatalyst and blue LED irradiation.^[17]^ This was (and remains) an extremely rare example of P_4_ successfully being transformed in a catalytic manner, and was uniquely capable of directly furnishing P−C bonds (Scheme 2a).

The reaction proceeds through the use of photoredox catalysis (PRC), a synthetic methodology that has had a dramatic impact on organic chemistry in recent years but has yet to be significantly applied to challenges in inorganic chemistry.[^18^, ^19^] The PRC mechanism involves photoreduction of the Ir‐based photocatalyst via initial photoexcitation and electron transfer from Et_3_N (which acts as the reaction's terminal reductant) to the resulting catalyst excited state (Scheme 2b). Thus reduced, the catalyst is able to effect one‐electron reduction of the aryl iodide substrate, regenerating the initial catalyst and producing an aryl radical upon extrusion of iodide. These radicals are then proposed to add directly to the P−P bonds of P_4_, generating in turn ArPH_2_, Ar_2_PH, Ar_3_P and (in some cases) Ar_4_P^+^. The extra H atoms in the intermediate structures are proposed to derive from the Et_3_N reductant (most likely via H⋅ or H^+^ abstraction following oxidation to Et_3_N^+.^), and this mechanism has been supported by significant mechanistic studies both in the initial publication and more recent follow‐up reports.^[20]^ Selectivity between Ar_3_P and Ar_4_P^+^ in the final product mixture is determined by electronic and (particularly) steric effects, with the tertiary phosphine being favoured by bulky and/or electron‐deficient arenes (Scheme 2c).

The use of radical mechanisms for the conversion of P_4_ into P_1_ products is relatively uncommon. However, specific examples have been described previously.^[21]^ In particular, in 2010 Cossairt and Cummins reported that radicals produced by halogen atom abstraction from organic (and some heteroatomic) precursors are able to add to P_4_ in a similar manner, even ultimately furnishing the corresponding tertiary monophosphines.^[22]^ This procedure suffered from the need for (super)stoichiometric quantities of a sensitive, high‐molecular‐weight Ti^III^ species (although this could be regenerated through Na/Hg reduction of the reaction byproduct), but can be considered an important conceptual precursor to the photochemical procedure described by Wolf et al.

**Scheme 3:**
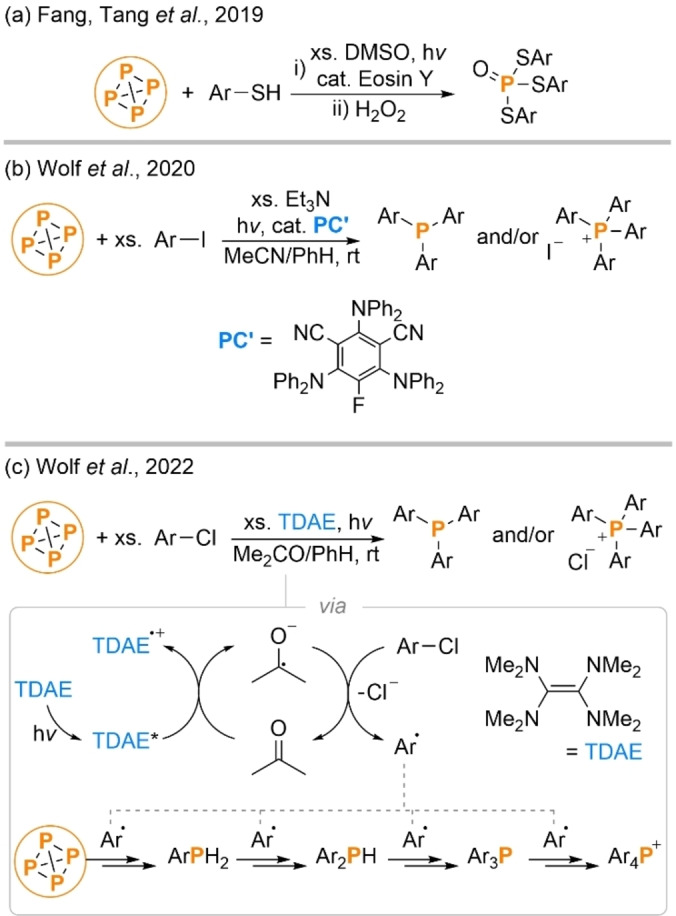
Studies conceptually related to Wolf et al.’s 2019 report have described photoredox‐catalysed oxidative thiolation of P_4_ (a), arylation of P_4_ using aryl iodides and an organic photoredox catalyst (b), and arylation of P_4_ using aryl chlorides and a stoichiometric photoreductant (c).

Even compared to the later systems described in this Minireview, the ability of this procedure to achieve fully catalytic P_4_ functionalization stands out, and its ability to form aryl C−P bonds allows direct access to products of high value to both industry and academia. Perhaps equally exciting is the proof of concept that photoredox methods can be used for P_4_ functionalization. While the authors describe only a specific reaction (arylation of P_4_ via aryl radicals derived from aryl iodides), PRC more broadly is known to provide access to a much wider range of radical intermediates from a much broader range of chemical precursors, any of which might feasibly be trapped by P_4_ in an analogous way.^[17]^ This suggests that this strategy could be significantly generalized. Indeed, around the same time as Wolf's report it was disclosed by Fang, Tang et al. that photoredox methods can also be used to effect the net oxidation of P_4_ to trithiophosphates (Scheme 3a).^[23]^ More recently, the Wolf group have reported that the iridium catalyst used in their initial publication can be replaced with a much cheaper organic photocatalyst (albeit at the cost of slightly reduced yields; Scheme 3b),^[24]^ and also that highly analogous arylation of P_4_ can be achieved using a stoichiometric photoreductant and cheap and abundant aryl chlorides as precursors (Scheme 3c).^[25]^ Each of these results points towards the generalizability of the underlying mechanistic concept.

### 2.2 Hydrostannylation of P_4_

**Scheme 4:**
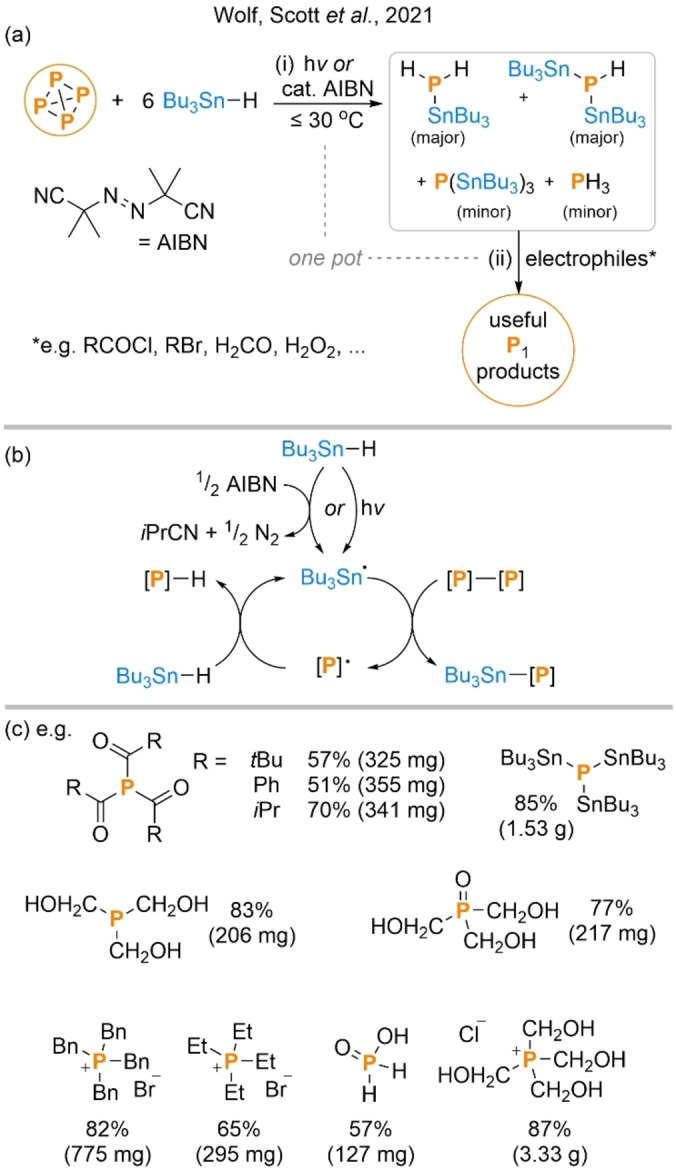
“One pot” hydrostannylation and subsequent electrophilic functionalization of P_4_ (a), proposed radical chain mechanism for hydrostannylation (b), and isolated products (c). Products observed but not isolated are not shown. [P]−[P] represents a generic P−P bond derived from P_4_.

Less than a year and a half after Wolf's disclosure of PRC‐mediated P_4_ functionalization a new report was published by Scott and Wolf describing a second, alternative method for the direct transformation of P_4_ into P_1_ products.^[26]^ Like the first, this new system also exploited the ability of radical methods to efficiently break down the P_4_ tetrahedron. However, whereas the earlier report had relied upon a relatively elaborate photochemical strategy for generation of the requisite radical intermediates, in this case a far simpler method was employed. Specifically, the authors reported that reaction of P_4_ with six equivalents of the classical radical reagent Bu_3_SnH in the presence of a suitable radical initiator (either a chemical initiator such as azobis(isobutyronitrile), AIBN, or irradiation with visible light) leads to complete breakdown of the P_4_ tetrahedron through what appears to be a simple but highly efficient radical chain process (Scheme 4a, b). The reaction was found to proceed under very mild conditions in a variety of solvents, and the 6 : 1 reaction stoichiometry reflects the need for one equivalent of the tin hydride for each of the six P−P bonds.

The immediate products of the reaction are the stannylated phosphine derivatives (Bu_3_Sn)_*x*_PH_3−*x*_ (*x*=0–3), with the main products being the partially substituted derivatives Bu_3_SnPH_2_ and (Bu_3_Sn)_2_PH, and the other products (PH_3_ and (Bu_3_Sn)_3_P) formed only in smaller amounts. The major products could be separated by distillation at high temperature and under high vacuum, and then individually reacted further. However, because each component functions as a chemically‐similar “P^3−^” synthon the crude (Bu_3_Sn)_*x*_PH_3−*x*_ mixture could also be functionalized directly, yielding single P_1_ products in a “one pot” fashion upon treatment with a variety of different classes of electrophiles (Scheme 4c). Reported products include highly industrially relevant compounds such as tetrakis(hydroxymethyl)phosphonium chloride (THPC; a flame‐retardant precursor) and were isolable in generally good to excellent yields, including for some examples at gram scale.

**Scheme 5:**
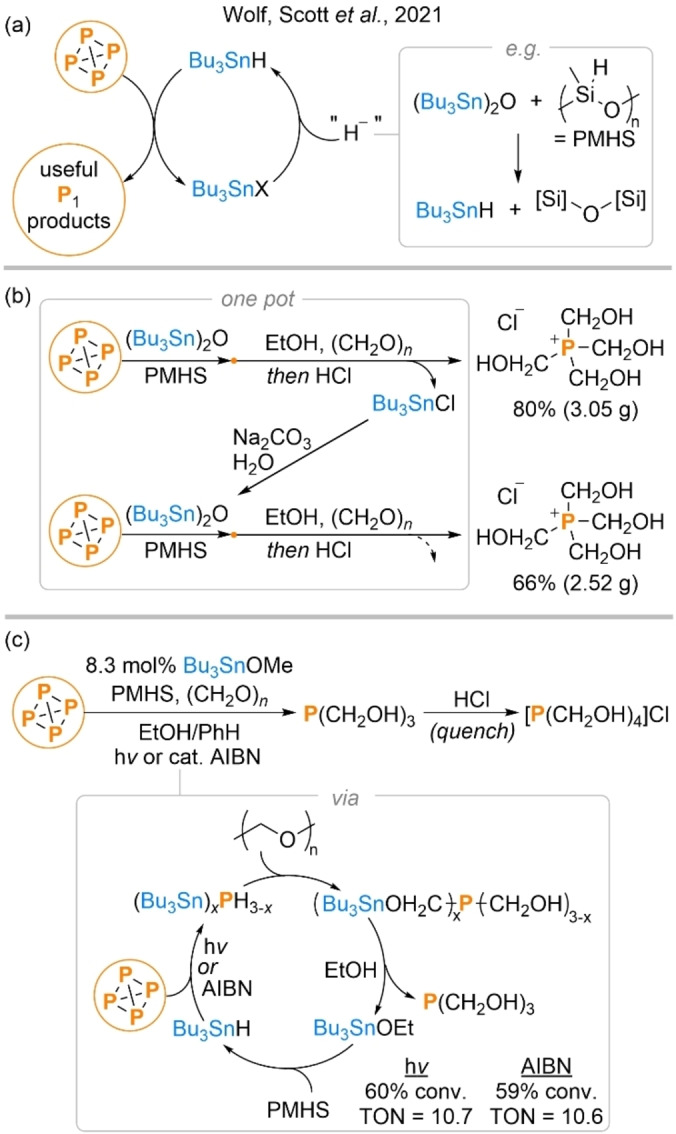
Closed loop strategy for hydrostannylative functionalization of P_4_ without net consumption of Bu_3_SnH (a), realisation of this closed loop in a “one pot” fashion (b), and catalytic functionalisation of P_4_ to THPC via P(CH_2_OH)_3_ (c). Catalyst loading in mol % is defined per P atom and turnover number (TON) per P−P bond.^[27]^

Formed alongside the target products of these reactions were byproducts of the type Bu_3_SnX (X=halide, ^1^/_2_ O, or alkoxide), which could be isolated in near‐quantitative yield during the workup of certain reactions. Since such compounds are known precursors for the synthesis of Bu_3_SnH, this allowed for the construction of a “closed loop” strategy in which the key tributyltin reagent could be recycled (Scheme 5a). Moreover, it was shown that this entire cycle could be performed in a “one pot” fashion (Scheme 5b), and that it was even possible to loop through the entire cycle twice without ever isolating or purifying the tin‐containing intermediates (although this did result in a drop in yield during the second cycle).

Extending this concept of a closed loop to its logical extreme, the authors were ultimately able to show that the Bu_3_SnH mediator could even be employed in a fully catalytic manner during the synthesis of THPC, achieving turnover numbers above ten (Scheme 5c).^[27]^ Despite the precedent previously set by Wolf's PRC method, this remains one of extremely few examples of catalytic P−C bond formation from P_4_. Notably, it also results in formation of P−C(sp^3^) bonds, in contrast to the former's exclusive formation of P−C(sp^2^) bonds. More generally, this hydrostannylation‐based procedure is notable for its simplicity (both conceptually and practically), for its corresponding versatility (allowing for access to structurally distinct product types), and for its use of cheap and readily available reagents (especially where the tributyltin moiety can be recycled).

### 2.3 Pentaphosphaferrocene‐Mediated Synthesis of Tertiary Phosphines

Just six months after Scott and Wolf's report of P_4_ hydrostannylation a third major breakthrough was reported by the Scheer group, who are longstanding contributors to the field of fundamental P_4_ activation and functionalization chemistry.^[28]^ While the two systems discussed so far exploited relatively underexplored radical methods, this new contribution was instead based upon the much more established concept of transition‐metal‐mediated P_4_ activation. Indeed, at the core of the reported method is the oligophosphorus‐containing ferrocene analogue Cp*FeP_5_, which has been studied extensively since it was first reported by Scherer in 1987, and can be prepared by direct reaction of [Cp*Fe(CO)_2_]_2_ with P_4_.^[29]^

**Scheme 6:**
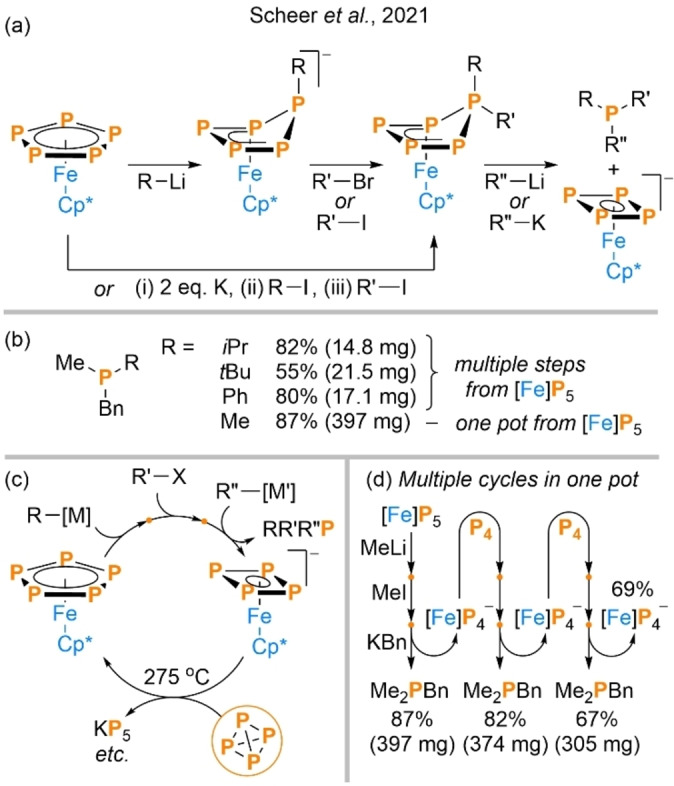
Synthesis of tertiary phosphines from Cp*FeP_5_ ([Fe]P_5_) (a), isolated products (b), closed loop for regeneration of [Fe]P_5_ from P_4_ (c), and demonstration of this cycle in “one pot” (d). For Me_2_PBn, masses are calculated from reported yields.

Building upon previous results that had shown the potential for selective nucleophilic addition to the *cyclo*‐P_5_ ring of this complex (including by organometallic reagents, RM),^[30]^ the authors were able to demonstrate that subsequent addition of an electrophile, R′X, can result in a second functionalization of the same phosphorus atom (Scheme 6a). The same net transformation could also be achieved by initial reduction of Cp*FeP_5_ using K metal, followed by addition of electrophiles. Further addition of a second organometallic nucleophile, R′′M′, then leads to a third addition to the same atom which is cleaved from the P_5_ ring, forming the corresponding free tertiary phosphine, RR′R“P. Notably, and in contrast to the previous systems discussed, the use of three distinct addition steps allows for the controlled installation of distinct substituents R/R′/R′′, and hence the convenient synthesis of phosphines with relatively complex substitution patterns (R≠R′≠R′′) that would otherwise be awkward to access. Moreover, despite the multiple reaction steps involved, the authors were able to demonstrate that the entire synthesis of a representative phosphine product could be performed in ”one pot“ from Cp*FeP_5_ by simple, sequential addition of the relevant reagents, without any need for intermediate purification, solvent switching or so on (Me_2_PBn; isolated in 87 % yield; Scheme 6b).

The organometallic byproduct of this net reaction is the anionic *cyclo*‐P_4_ complex [Cp*FeP_4_]^−^. Remarkably, in order to avoid this material being lost as stoichiometric waste, the authors were able to demonstrate its conversion back into the Cp*FeP_5_ starting material by heating with one equivalent of P_4_ at 275 °C (following convenient separation of the product Me_2_PBn from the crude mixture by distillation; Scheme 6c). Thus regenerated it could then, without purification, be exposed again to the same sequence of “one pot” reaction conditions, generating a second batch of Me_2_PBn in almost identical yield (82 % vs. 87 %) and providing a direct, cyclic route to this product from P_4_. Only upon subjecting the material to a third cycle was the yield appreciably reduced (to a still good 67 %; Scheme 6d). Like the hydrostannylation procedure discussed previously this strategy is notable for its versatility, with its elementary steps apparently being compatible with formation of P−C(sp^2^), P−C(sp^3^) and other P−E bonds and thus providing access to a potentially very wide variety of RR′R′′P products (although it should be acknowledged that the full sequence of steps was only reported for fully organic derivatives).

### 2.4 Electrochemical Cyanation of P_4_

The penultimate system discussed in this Minireview was reported just four months after that of Scheer and co‐workers.^[31]^ Similarly to that contribution, in their investigations Liu et al. sought to exploit the reactivity of a long‐known phosphorus‐containing species: the anion [P(CN)_2_]^−^, which was originally reported by Schmidpeter in 1977.^[32]^ In fact, the direct synthesis of [P(CN)_2_]^−^ salts from P_4_ had been reported before, through direct heating in the presence of KCN and 18‐crown‐6 (18c6).^[33]^ Unfortunately, the stoichiometry of this reaction results in very poor P atom economy, with 15 atoms being unproductively lost in the form of K(18c6)P_15_ for every one atom incorporated into the desired product. The authors therefore sought a more efficient, low‐waste synthesis of the same product using electrochemistry. While previous studies have investigated the use of electrochemical methods in the chemistry of P_4_, Liu et al. themselves noted that “*practical electrochemical activation of P_4_ is still in its infancy*”.^[34]^

**Scheme 7:**
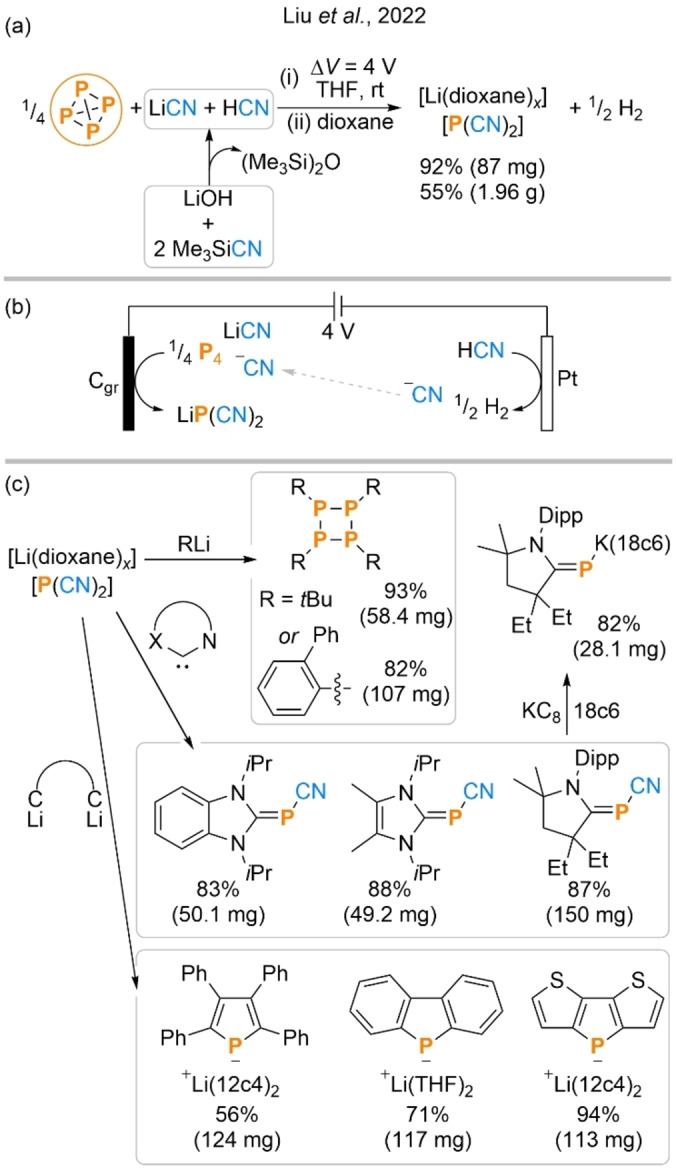
Electrochemical synthesis of [Li(dioxane)_*x*_][P(CN)_2_] directly from P_4_ (a), schematic of the electrochemical mechanism (b), and products generated from [Li(dioxane)_*x*_][P(CN)_2_] (c). Products observed but not isolated are not shown.

Despite this, the authors were successful in the development of their target reaction. By combining P_4_ with LiCN and HCN in an undivided electrochemical cell under a constant 4 V potential they were able to prepare the target anion in the form of the salt [Li(dioxane)_*x*_][P(CN)_2_] in excellent 92 % yield (albeit reduced to 55 % upon upscaling from 0.125 mmol to 5.0 mmol P_4_). In principle the reaction proceeds with excellent atom economy with H_2_ as the only byproduct, although in practice HCN and LiCN were generated in situ through combination of Me_3_SiCN and LiOH and elimination of (Me_3_Si)_2_O, to avoid direct use of extremely toxic HCN gas (Scheme 7a, b).

Strictly speaking, the product [Li(dioxane)_*x*_][P(CN)_2_] is the only product in this report to be *directly* prepared from P_4_ (besides the related Na and K salts, which could not be isolated in analytically pure form). Nevertheless, the authors were able to demonstrate that this salt is a useful precursor to other P_1_ compounds, generating phosphinidene, cyclophosphane and phospholide derivatives in good to excellent yields upon reaction with carbenes, organolithium and organodilithium reagents,^[35]^ respectively (Scheme 7c). The possibility of “one pot” preparation and subsequent functionalization of [P(CN)_2_]^−^ was unfortunately not discussed in this report. The inert nature of the stoichiometric byproducts would seem to suggest that such a protocol should be feasible, although it is possible that the presence of residual cyanide salts (which are used in excess in the optimized procedure) could have a deleterious impact on downstream reactivity.

### 2.5 Oxidative Onioation of P_4_

The final system under discussion was reported by the Weigand group very recently, and just three months after the contribution of Liu and co‐workers. Interestingly, this system can be considered a conceptual inversion of the hydrostannylation strategy employed by Scott and Wolf, and the two are thus highly complementary. Whereas the latter strategy was based around the reduction of P_4_ to a formal source of “P^3−^” followed by reaction with electrophiles, Weigand et al. have developed a formal oxidation of P_4_ to give a “P^3+^” synthon, which can then undergo reaction with nucleophiles.^[36]^

**Scheme 8:**
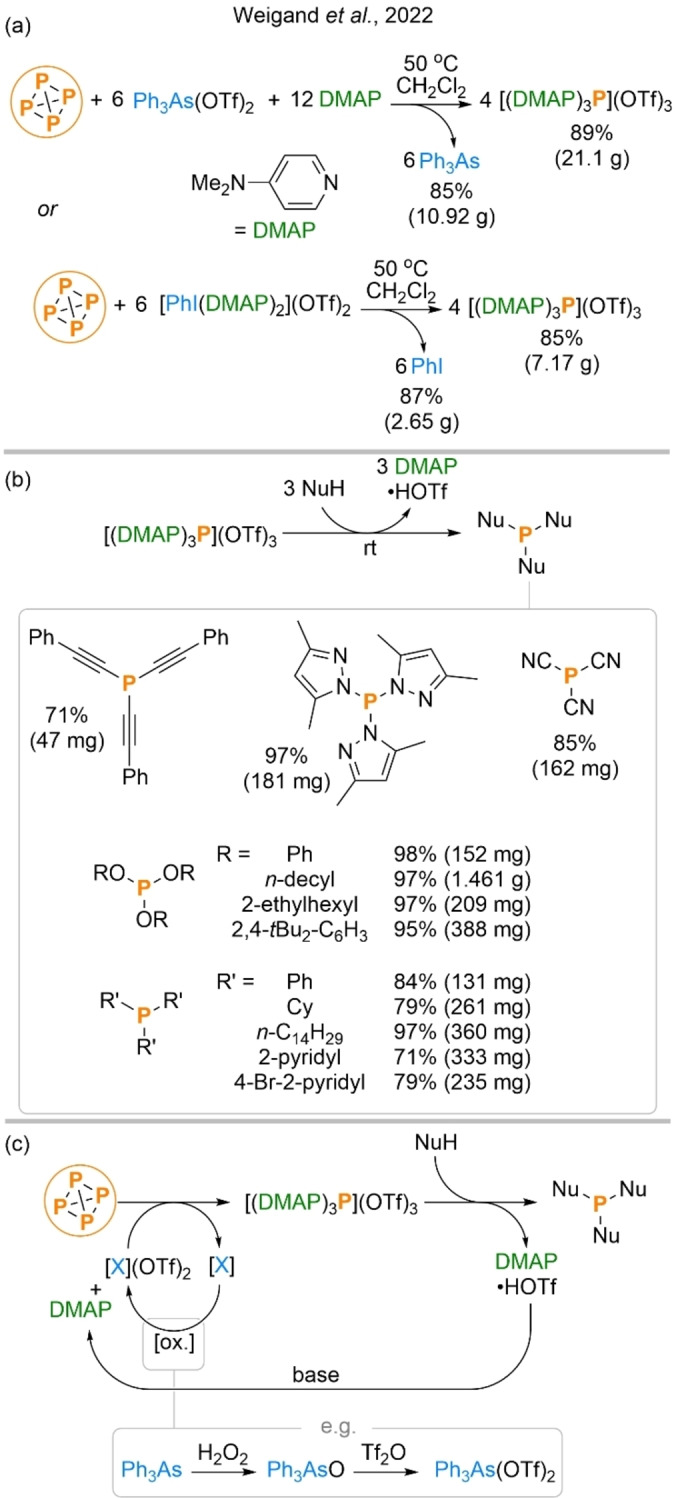
Oxidation of P_4_ to [(DMAP)_3_P]^3+^ (a), subsequent transformation into P_1_ products (b), and closed loop strategy for recycling of base and oxidant (c). Products observed but not isolated are not shown. To produce P(CN)_3_, Me_3_SiCN was used instead of HCN (and DMAP⋅Me_3_SiOTf was produced). To produce R′_3_P, R′MgX was used instead of R′H (and DMAP and “MgXOTf” were produced).

Specifically, the authors describe a strategy they term “*oxidative onioation*”, which relies on reaction of P_4_ with a suitable oxidizing Lewis acid and nitrogen‐centred Lewis base. Using Ph_3_As(OTf)_2_ and DMAP (4‐dimethylaminopyridine) was found to result in formation of the highly electrophilic trication [(DMAP)_3_P]^3+^ as a noncorrosive solid in excellent yield at multigram scale (Scheme 8a, top). The Ph_3_As byproduct of the reaction could also be cleanly isolated and used to regenerate the initial oxidant. Similar results were achieved using an iodine(III) reagent in place of Ph_3_As(OTf)_2_, with PhI as the isolable and recyclable byproduct (Scheme 8a, bottom).

Being effectively a base‐stabilised form of P^3+^, [(DMAP)_3_P]^3+^ was found to react readily with a variety of simple pronucleophiles, forming new P−O, P−N and even P−C bonds. Products include industrially relevant compounds such as phosphites and phosphines (including Ph_3_P), and are formed in typically excellent yields under mild conditions (Scheme 8b). In several cases it was shown DMAP‐based byproducts of these reactions (DMAP⋅HOTf or DMAP⋅Me_3_SiOTf) could also be recovered for potential recycling in excellent yields. Thus, both the oxidant and base used during P_4_ functionalization could potentially be used in a closed loop fashion via known chemistry (Scheme 8c).^[37]^

As for [P(CN)_2_]^−^, the possibility of synthesis and further transformation of [(DMAP)_3_P]^3+^ being achieved in a “one pot” fashion does not appear to have been pursued, and so strictly speaking the above products are not accessed directly from P_4_ (besides [(DMAP)_3_P](OTf)_3_ itself). Nevertheless, the simple byproducts (Ph_3_As or PhI), use of exact stoichiometry, and apparently very clean nature of the [(DMAP)_3_P]^3+^ functionalization step would seem to suggest that this should be feasible. Regardless, the system offers both an operationally and conceptually simple strategy for the synthesis of P_1_ products from P_4_. While the oxidants involved are somewhat elaborate, this limitation is mitigated by the potential recycling of their byproducts using commercial reagents and the system also appears to be highly versatile, being compatible with a wide variety of different (pro)nucleophiles.

### 2.6 Comparisons and Limitations

Despite the mechanistic diversity of the approaches used, it is interesting to note that the strategies employed by Scott/Wolf, Liu and Weigand are all broadly analogous, in that P_4_ is initially transformed into a simple P_1_ source which is then further transformed into the final target products. As such, it is interesting to compare the substrate scope of these methods. Doing so reveals that, while Wolf/Scott and Weigand have mainly used simple electrophilic or nucleophilic reagents to generate similarly simple but industrially relevant products, Liu et al. have prepared more esoteric structures of less direct industrial relevance, but significant academic interest.

A possible reason for this discrepancy can be found by considering the reactivity of the different systems’ P_1_ synthons. Scott and Wolf's (Bu_3_Sn)_*x*_PH_3−*x*_ mixture functions as a “P^3−^” source, while Weigand's intermediate behaves as “P^3+^”, capable of reacting in a simple manner with electrophiles and nucleophiles, respectively, to give products of the type “PX_3_”. In contrast, a combination of negative charge and CN^−^ leaving groups causes [P(CN)_2_]^−^ to act as a more unusual source of “P^+^”. Elaboration of this synthon into simple structures of the type “PX_3_” is more awkward, formally requiring both nucleophilic and electrophilic sources of “X” (in this regard there is an obvious similarity to Scheer's system, in which Cp*FeP_5_ also acts as a formal source of P^+^). Conversely, though, it allows convenient access to Liu and co‐workers’ reported products, which would be harder to access from P^3+^/P^3−^. In this sense, the work of Liu can be considered complementary to that of Wolf/Scott and Weigand, with each providing convenient access to different P_1_ products through the intermediacy of different P_1_ synthons. Indeed, understanding these P_4_ functionalisation reactions in these terms is likely to be beneficial when trying to identify direct strategies for the synthesis of further P_1_ targets in the future. As a simple, arbitrary example, while none of the methods described has yet been reported to provide direct access to simple hexaalkyl phosphorus triamides (R_2_N)_3_P, it can be anticipated that such compounds would most conveniently be prepared using nucleophilic precursors “R_2_N^−^” (e.g. R_2_NH) and would therefore require a corresponding P^3+^ synthon. Weigand's oxidative onioation strategy would thus be a promising starting point for such targets.

While discussion thus far has largely focused on the advantageous features of the systems being highlighted, it is important also to acknowledge their limitations. Despite the elegance of Wolf's PRC strategy, it can be recognized that for optimal results it employs reagents in significant excess, alongside an expensive precious metal catalyst. It also requires expensive aryl iodides as substrates (with neither other aryl halides nor alkyl substrates yet being compatible) and for many of these conversions are low, with isolated yields being further limited by difficult workups. More fundamentally, selectivity between Ar_3_P and Ar_4_P^+^ is often poor and is determined entirely by the substrate (leaving headline targets including Ph_3_P itself still out of reach), and recent follow‐up studies have emphasized the mechanistic complexity of the reaction, which leads to deleterious side‐reactivity and difficulties in rational reaction optimization.^[20]^

Scott and Wolf's hydrostannylation procedure is conceptually simpler but suffers from the toxicity of its organotin derivatives, which is only partially mitigated through the use of “closed loop” strategies. The generation of a (Bu_3_Sn)_*x*_PH_3−*x*_ mixture rather than a single intermediate also complicates reaction development, and it is likely that formation of gaseous PH_3_ as a minor component of this mixture has a limiting effect on overall yields. Moreover, while the procedures are “one pot” they usually involve multiple steps, and although catalysis has been demonstrated this is currently limited to a single example.

Similarly, Scheer's iron‐mediated process requires multiple steps, even when performed in “one pot” fashion. At various stages it also employs extreme temperatures and sensitive and mutually incompatible reagents, suggesting that progress towards simplified or catalytic procedures is likely to be challenging. Perhaps most importantly, the overall procedure has very poor P atom economy, with 75 % of the P_4_‐derived atoms being lost as KP_5_ and related polyphosphides, unless stoichiometric amounts of [Cp*FeBr]_2_ are also added to transform these into Cp*FeP_5_.

Liu's electrochemical strategy again suffers from the use of highly toxic reagents (cyanides), and has not yet been shown either to give *direct* access to useful products beyond the [P(CN)_2_]^−^ salts themselves, or to provide access to particularly industrially (rather than academically) relevant motifs. Finally, Weigand's oxidative onioation again requires at least a two‐reaction sequence from P_4_, and also requires stoichiometric use of relatively elaborate reagents. While these can in principle be recycled, recovering both the oxidant and base adds significant practical complexity alongside several additional reaction steps.

## 3 Summary and Outlook

The breakthroughs that have been described in this Minireview remain in their early stages, and significantly more research will clearly be needed if any of them is to truly threaten the current industrial state of the art. This, however, cannot obscure the remarkable progress that has been made in the last two and a half years, which has seen direct P_4_ functionalization transition sharply from a mere future aspiration to a proven reality.^[38]^ Each system discussed represents a compelling proof of principle that we can expect to see further developed, broadened and refined in subsequent reports. In fact, clear evidence of such progress can already be seen for the earliest of these systems, with Wolf's PRC method already having been adapted to use an organic catalyst,^[24]^ had its mechanism studied in deeper detail,^[20]^ and seen its underlying concept extrapolated to allow activation of more industrially viable aryl chlorides.^[25]^

From the examples set by these systems, we can tentatively begin to identify priorities for the future field of P_4_ functionalization. First, despite the mechanistic novelty displayed by most of the highlighted reports, in most cases a clear link can be drawn back to previous, more fundamental studies on the reactivity of P_4_.[^21^, ^22^, ^29^, ^30^, ^33^] Thus, the need for continued study of elementary P_4_ reactivity should certainly not be dismissed. Nevertheless, these results also clearly show that moving beyond simple P_4_ “activation”—and even indirect functionalization—is no longer an overambitious goal. Direct transformation should therefore become an increasingly dominant target.

**Scheme 9:**
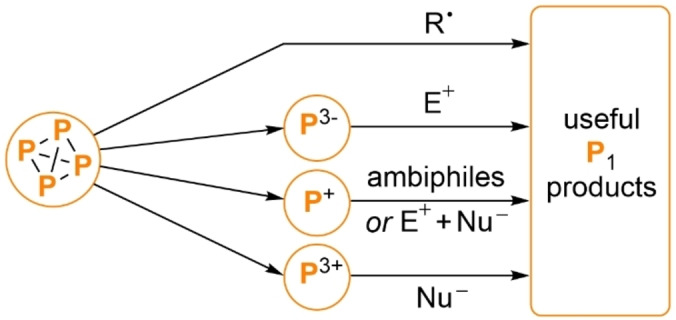
Schematic summary of the different strategies employed for P_4_ functionalization in the reports highlighted in this Minireview: direct radical addition (Wolf), or intermediate generation of “P^3−^” (Scott/Wolf), “P^+^” (Scheer, Liu) or “P^3+^” (Weigand) synthons followed by further reactions.

A second conspicuous factor has been the mechanistic diversity of these recent reports. Of the five articles discussed, only one has involved transition metal polyphosphorus intermediates, with the remainder instead exploiting techniques as varied as photocatalysis, electrochemistry and classical radical chain reactivity. This suggests that emphasis should be placed on understanding and exploiting the reactivity of P_4_ towards a much broader range of reagents than currently. Indeed, the results to date further illustrate the benefits of pursuing a diverse range of synthetic strategies, as by providing access to chemically distinct “P^3+^” (Weigand), “P^+^” (Scheer, Liu), “P^0^” (Wolf)^[39]^ and “P^3−^” (Scott/Wolf) synthons they each allow access to products that would be difficult to prepare by the other methods (Scheme 9).

Finally, with the goal of eventual industrial relevance in mind, future efforts should whenever possible aim to use cheap terminal reagents, to employ benign chemical mediators, and to produce minimal waste—a set of goals that has certainly not been fully met by any of the highlighted systems. As a means towards this, specific emphasis should continue to be placed on the pursuit of new, *catalytic* P_4_ functionalization processes, which remain exceedingly rare.

Ultimately, the results that have been discussed in this Minireview show that the field of P_4_ functionalization has by now achieved an advanced level of understanding, sufficient to move beyond the study of purely elementary reactivity to target ever more ambitious, direct and industrially relevant transformations. The ability to directly convert P_4_ into useful P_1_ products by not just one but a range of mechanistically distinct synthetic strategies suggests the start of a new phase in P_4_ research, and their appearance within such a brief period of time offers the promise of many more such exciting breakthroughs in the years to come.

## Biographical Information

*Daniel Scott earned his PhD from Imperial College London (UK) under the supervision of Dr. Andrew Ashley and Prof. Matthew Fuchter. He subsequently received an EPSRC Doctoral Prize fellowship at the same institution, followed by an Alexander von Humboldt Fellowship at the University of Regensburg to work with Prof. Robert Wolf. He is currently an EPSRC Early Career Fellow at the University of Oxford, where his research interests include synthetic photochemistry and the activation and functionalization of industrially significant small molecules*.